# Dual-targeted nanotechnology for gynaecological cancers: a comprehensive review of mechanisms and clinical potential

**DOI:** 10.1080/07853890.2026.2738261

**Published:** 2026-10-04

**Authors:** Xinyun Tu, Youni Zhang, Li Wang, Yiyi Shan, Lihong Xie, Huafeng Shou, Yan Liang

**Affiliations:** a Aksu First People’s Hospital, Aksu, China; b Department of Laboratory Medicine, Tiantai People’ s Hospital, Taizhou, China; c Center for Reproductive Medicine, Department of Gynecology, Zhejiang Provincial People’s Hospital (Affiliated People’s Hospital, Hangzhou Medical College), Hangzhou, China; d Aksu Maternal and Child Health Hospital, Aksu, China

**Keywords:** Dual-targeted nanotechnology, gynaecological cancer treatment, targeted drug delivery systems, nanoparticle-based therapy, clinical applications of nanotechnology

## Abstract

**Background:**

Ovarian, cervical and endometrial cancers have been the leading cause of morbidity and mortality in women, often resulting from late diagnosis, resistance to therapies and off-target toxicity of conventional therapies.

**Discussion:**

The review discusses recent progress in the development of dual-targeted nanocarrier systems in gynaecologic oncology, relying on peer-reviewed publications in scientific databases published within the past decade. Applications of these systems are also discussed in terms of the potential to improve the selectivity and therapeutic efficiency of tumour therapy. Major available evidence is based on preclinical *in vitro* and *in vivo* models. Clinical translation is in its early-phase trials, suggesting clinical validation in gynaecologic oncology remains in its infancy. The most important challenges in translation are scalable manufacturing, long-term safety, biological complexity and regulatory challenges.

**Conclusion:**

This review describes the prevailing situation and constraints of dual-targeted nanotechnology, focusing on possible applications in the development of more accurate and individualized therapies for gynaecologic cancers.

## 1. Introduction

Gynaecologic cancers, originating in reproductive organs such as the cervix, ovaries, uterus, vagina and vulva, remain a significant global health concern for women. These malignancies account for approximately 16.5% of the projected 8.2 million new cancer cases in women worldwide this year, highlighting their considerable impact on female health [1]. Cervical cancer (CC) is primarily associated with persistent infection by the human papillomavirus and is largely preventable through vaccination and regular cervical screening [2]. Ovarian cancer is often diagnosed at an advanced stage due to its nonspecific early symptoms, contributing to its high mortality rate. Despite advances in targeted therapies, it remains one of the most lethal gynaecologic malignancies [3]. Endometrial cancer is the most frequently diagnosed gynaecologic malignancy in the United States and is strongly linked to obesity and hormonal imbalances. Although vaginal and vulvar cancers are less prevalent, they are frequently diagnosed at later stages and present limited therapeutic options. Epidemiological assessment of these cancers is essential for the development of effective early detection frameworks and therapeutic interventions, both of which are pivotal in improving clinical outcomes among affected women [3].

Current treatment approaches for gynaecological cancers face significant challenges that hinder their efficacy and negatively impact patients’ quality of life. A major limitation is the low specificity of therapies, which often leads to severe side effects. For instance, chemotherapy commonly used for ovarian cancer and CC is associated with adverse haematological, gastrointestinal and dermatological toxicities that can diminish patient well-being during and after treatment [4]. Additionally, patient adherence to chemotherapy regimens is often poor; studies report that over 70% of ovarian cancer patients do not complete their prescribed treatments due to debilitating side effects and logistical challenges such as travel to treatment centres. Surgical options, although sometimes necessary, carry high risks and are linked to considerable postoperative complications. like elevated postoperative mortality and long-term issues such as bladder dysfunction and lymphedema [5]. Innate and acquired chemoresistance, along with strong intra- and inter-tumoral heterogeneity, continue to be the primary contributors to failure of treatment in gynaecologic cancers, in particular, ovarian cancer and recurrent CC [6].

Manipulation of matter at the nanoscale (1–100 nm), known as nanotechnology, has emerged as a promising strategy in cancer research and treatment. It facilitates the targeted delivery of therapeutic agents to malignant cells, which may improve efficacy while reducing systemic toxicity. Clinicians can achieve improved drug delivery through the availability of Nanoparticles (NPs), enhanced permeability and retention (EPR) effect, which enables delivering higher concentrations of therapeutic agents to tumour areas, as highlighted in Figure 1. Although preclinical models consistently demonstrate two- to sixfold higher accumulation in tumours versus normal tissue [7,8]. Clinical studies reveal significant heterogeneity across tumour types and patients. For instance, retrospective analyses of clinical trials show variable NP uptake influenced by tumour histology, size and perfusion [9]. Yet, nanotechnology accelerates the development of improved diagnostic techniques that can lead to early detection of tumours and tracking the progress of patient during his treatment [10]. Nanotechnology is advancing diagnostic capabilities. Radiolabelled PEGylated liposomes in phase I/II clinical studies demonstrated tumour accumulation detectable by scintigraphic imaging—tumours were visualized in patients up to one week post-administration [11]. Moreover, NP-based contrast agents, such as iron oxide nanocluster and gold nanocluster (AuNC), are being investigated for enhanced magnetic resonance imaging (MRI) sensitivity and theranostic applications in early detection and treatment monitoring [12].

**Figure 1.**
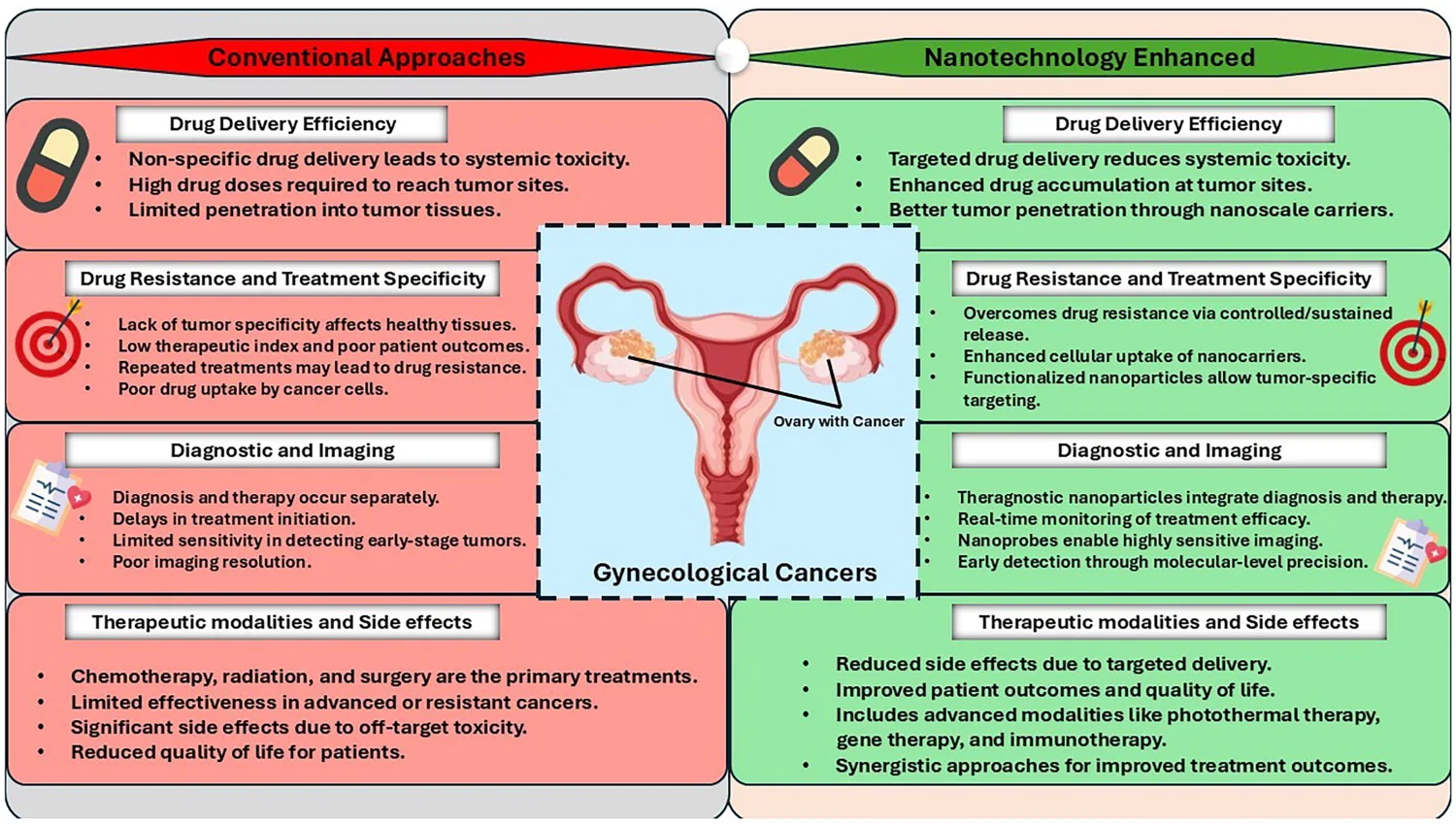
Schematic representation of emerging nanotechnology-based approaches for the diagnosis, treatment and management of gynaecologic cancers. These strategies aim to improve drug delivery, overcome therapeutic resistance, enhance imaging precision and reduce systemic side effects. While many of these mechanisms have demonstrated promise in preclinical studies, their clinical translation is still under investigation. The figure is conceptual and is created by the authors based on current research findings.

To counter the biological complexity of gynaecologic tumours, dual-targeted nanocarrier systems have emerged. Simultaneous targeting of tumour cells and tumour-associated features, including angiogenic vasculature, cancer stem cells and tumour microenvironment (TME), these dual-targeted systems overcome chemoresistance, receptor heterogeneity and limited intratumoural drug penetration [13]. While these benefits have been demonstrated in numerous preclinical studies, clinical translation remains limited to a subset of approved formulations, and further trials are ongoing to establish broader clinical efficacy [14]. For example, attachment of certain peptides to NPs greatly enhances the targeting of drugs such as doxorubicin (DOX) to cancer stem cells as well as bulk tumour cells and also reduces drug resistance and increases survival in preclinical models [13]. This review opens by covering approaches to nanotechnology in gynaecological cancer, which extend beyond passive targeting to active targeting. Further, it outlines targeting designs in detail. Various dual-targeting strategies are addressed, such as one where a single-ligand targets a common receptor on various cellular constituents, and the one where two different ligands target different receptors on different tumour-related cells. In the next sections, the benefits of dual-target systems are discussed, followed by the underlying mechanism of the dual-targeted nanotechnology, tumour-targeting pathways and synergistic therapeutic effects. Recent clinical practice is discussed with a focus on ovarian cancer, CC and other malignancies associated with gynaecology. Finally, issues about biocompatibility, toxicity, clinical translation, manufacturing and scale-up are discussed, followed by future perspectives.

## 2. Nanotechnology in gynaecological cancers

In nanomedicine, targeting strategies are designed to improve drug delivery precision through the use of tumour-specific properties and molecular markers to achieve better therapeutic outcomes with minimum side effects.

### 2.1. Passive targeting

The phenomenon of preferential accumulation of certain macromolecules in tumours was first observed over 30 years ago [15]. The initial report of tumour-targeting therapeutic macromolecules was associated with poly(Styrene-co-Maleic Acid)-NeoCarzinoStatin (SMANCS), a 16 kDa polymer conjugate that non-covalently binds to albumin in circulation, resulting in an effective molecular weight of approximately 80 kDa [16,17]. Early preclinical studies revealed that SMANCS preferentially accumulated at tumour sites, leading Matsumura and Maeda to further investigate this phenomenon. By employing labelled albumin and other proteins in conjunction with the polymer conjugate, they demonstrated that proteins exceeding 30 kDa—including SMANCS, murine albumin (67 kDa), bovine albumin (69 kDa) and immunoglobulins (160 kDa)—selectively localized within the tumour interstitium and persisted for extended durations, up to 144 h. The EPR effect is a well-established mechanism that facilitates the selective accumulation of therapeutic agents in tumour tissues. This phenomenon arises due to the presence of abnormally large fenestrations in the tumour vasculature, which allow macromolecules and NPs to leak into the tumour interstitium passively [18,19]. In parallel, tumours often lack efficient lymphatic drainage, preventing the clearance of these agents and promoting their retention within the tumour site. Collectively, these features enable enhanced localization of drug-loaded carriers, such as polymer conjugates, liposomes and NPs at the tumour site. When a solid tumour reaches a certain size, the normal vasculature surrounding it becomes inadequate to supply the necessary oxygen for continued proliferation [20].

Dying tumour cells release growth factors that promote angiogenesis—the formation of new blood vessels from adjacent capillaries. This process leads to the rapid development of abnormal, irregular vasculature characterized by discontinuous endothelium and absence of a basal membrane, features atypical of normal blood vessels [21]. The size of fenestrations within these tumour-associated capillaries varies between 200 and 2000 nm, depending on the tumour type, its microenvironment and anatomical location. Consequently, blood components passing through this abnormal vascular network encounter minimal resistance, facilitating their extravasation into the tumour interstitium [22]. In normal tissues, extracellular fluid is continuously drained into lymphatic vessels at an average flow velocity of approximately 0.1–2 μm/s [23]. This process facilitates the ongoing renewal of interstitial fluid and the recycling of extravasated solutes and colloids back into circulation [24]. In tumours, impaired lymphatic function often limits the uptake of interstitial fluid, preventing colloids from using convective forces to return to the bloodstream. While molecules smaller than 4 nm can diffuse back into circulation, the diffusion of larger macromolecules or NPs is hindered by their increased hydrodynamic size. As a result, NPs that penetrate the perivascular space are inefficiently cleared and consequently accumulate within the tumour interstitium [25,26]. In solid tumours, molecular distribution is governed by three interconnected processes: extravasation of colloids from blood vessels, diffusion through the extravascular tissue and interactions with intracellular or extracellular targets within the TME. The first two processes are driven by diffusion and convection, influenced by tumour biology and the properties of the diffusing molecules. The third process—encompassing adsorption, cellular uptake and metabolic degradation—is less understood but critically affects substance accumulation. This complex interplay depends on factors such as material properties, affinity for tissue components and the tumour’s composition, including the extracellular matrix (ECM) and resident cell types. Due to its intricacy, these interactions are often described as ‘black box’ [27].

### 2.2. Active targeting

Ligand-mediated, or active targeting, is crucial for delivering drugs, genes and theranostic agents specifically to diseased sites while minimizing exposure to healthy tissues. This strategy involves decorating NP surfaces with affinity ligands that selectively bind to receptors overexpressed on target cells, thereby enhancing therapeutic efficacy and reducing off-target effects. For active targeting to be effective, NPs must first localize near the target cells, promoting stronger interactions and facilitating cellular internalization without significantly altering systemic biodistribution [28]. This strategy has proven effective through decoration of the surface of nanocarrier with ligands binding specifically to tumour cell receptors, aiding the penetration of drug into the TME. The incorporation of this phenomenon was first evidenced in 1980 using antibodies grafted onto liposomes [29], and then by grafting other ligands such as peptides, nucleic acids and aptamers [30,31].

Transferrin receptors (TfR) and nicotinic acetylcholine receptors present the classical targets for drug delivery into the brain TME. The primary means of this mechanism is vascular targeting of the endothelial cells. In the area of glioma treatment, transferrin ligands have been conjugated to different nanocarriers, such as solid lipid nanoparticles (SLNs) [32], micelles [33], dendrimers [34,35] and superparamagnetic iron oxide NPs. Furthermore, micelles have been able to target the CNS and glioblastoma using nicotinic acetylcholine receptor interactions [36,37]. Many receptors have been identified with corresponding antibodies successfully synthesized and evaluated both *in vitro* and *in vivo*, demonstrating strong ligand–receptor interactions that enhance active targeting. For example, the RGD peptide effectively promotes binding to the αVβ3 integrin, which is expressed on glioma cells and diseased endothelium [38]. Additionally, the F3 peptide targets the nucleolin receptor expressed on angiogenic endothelial cells within the TME. Aminopeptidase N (CD13) has also been identified as a promising receptor target in the TME [39], with a tri-peptide (Asn–Gly–Arg, or NGR) identified as its targeting agent. Among classical ligands, folic acid (FA) specifically binds to the folate receptor (FAR), which is also present in the TME. Various strategies have been reported to enhance drug delivery through FA-drug conjugates and FA grafting onto nanocarriers, promoting their endocytosis by cancer cells [40].

While both passive and active targeting strategies have shown promise in preclinical models, several comprehensive reviews have highlighted that *in vivo* tumour accumulation of NPs remains generally low, often averaging less than 1% of the injected dose. This discrepancy between theoretical targeting efficiency and actual delivery performance is influenced by numerous physiological barriers, including tumour heterogeneity, high interstitial pressure, rapid clearance by the mononuclear phagocyte system (MPS) and variability in vascular permeability across tumour types. These findings suggest the need for ongoing refinement of nanocarrier designs and a better understanding of the TME to improve clinical translation [9,41,42].

### 2.3. Dual-targeting strategies

Dual targeting enhances NP delivery efficiency to cancer cells by combining biological targeting with physicochemical-responsive smart delivery strategies. This approach increases NP accumulation within the TME. It involves either using a single ligand to target one receptor on specific cells or employing two different ligands to target two distinct receptors on the same or different cells [14] (Figure 2). In recent developments within gynaecologic oncology, dual-targeted drug delivery systems (DDSs) have shown significant promise over traditional mono-targeted approaches. These advanced systems utilize two targeting strategies simultaneously for instance, receptor binding and intracellular environment responsiveness to improve drug accumulation specifically within tumour cells. One notable example involves FAR-targeted ZIF-90 NPs carrying cisplatin, which are also responsive to pH and ATP levels. This dual mechanism not only enhances mitochondrial targeting in resistant ovarian cancer cells but also improves therapeutic efficiency when compared to single-target carriers [44]. Likewise, researchers have developed NPs combining DOX with Bcl-2 siRNA, designed to bind FARs and silence resistance-related genes. These particles significantly boost apoptosis in ovarian cancer stem cells, showing synergistic effects that single-mode treatments often fail to achieve. Overall, dual-targeted platforms offer a more precise and robust therapeutic strategy by addressing both targeting and resistance simultaneously, making them highly relevant for real-world clinical applications [45]. Dual-targeted mesoporous silica nanoassembly functionalized with folate and epidermal growth factor receptors (MSN@Px-L-GF) exhibited a quantitative enhancement in therapeutic effect, which reached close to an eight-fold enhancement in anticancer activity. This was found to be more effective due to a better receptor-mediated uptake in the cells through folate and EGFR targeting, a higher clathrin-mediated endocytosis and pH-dependent release of drugs in the TME. The system also caused apoptosis by damaging mitochondria (reactive oxygen species [ROS]-mediated), which emphasizes the importance of dual-targeting strategies in preclinical models profited compared with non-targeted or single-targeted strategies [46]. T7-modified magnetic PLGA NPs containing paclitaxel and curcumin co-encapsulated showed a more than tenfold increase in cellular uptake and more than five-fold increase in delivery into the brain. Dual targeting in an orthotopic glioma model allowed the efficient accumulation of the brain in the brain, which is proven by fluorescence, synchrotron radiation X-ray and MRI. The system was found to be significantly better therapeutically, with 100% of glioma-bearing mice surviving post-treatment, versus 62.5 by using the free drug combination, indicating the quantitative benefit of dual-targeted therapies over others [47]. Non-targeted formulations (PDA-DEM and PDA-DEM-Fe_3_O_4_) showed limited inhibition, with cell viabilities of 90% and 88%. Co-targeting enhanced cellular accumulation and improved efficacy, reducing cell viability to ∼82%. When combined with synergistic photothermal and chemotherapeutic effects, co-targeted systems achieved a marked reduction in cell viability up to 19% as compared with 48–54% for non-co-targeted systems. These findings illustrate superior performance of dual-targeted approaches over single-targeted or non-targeted strategies [48].

**Figure 2.**
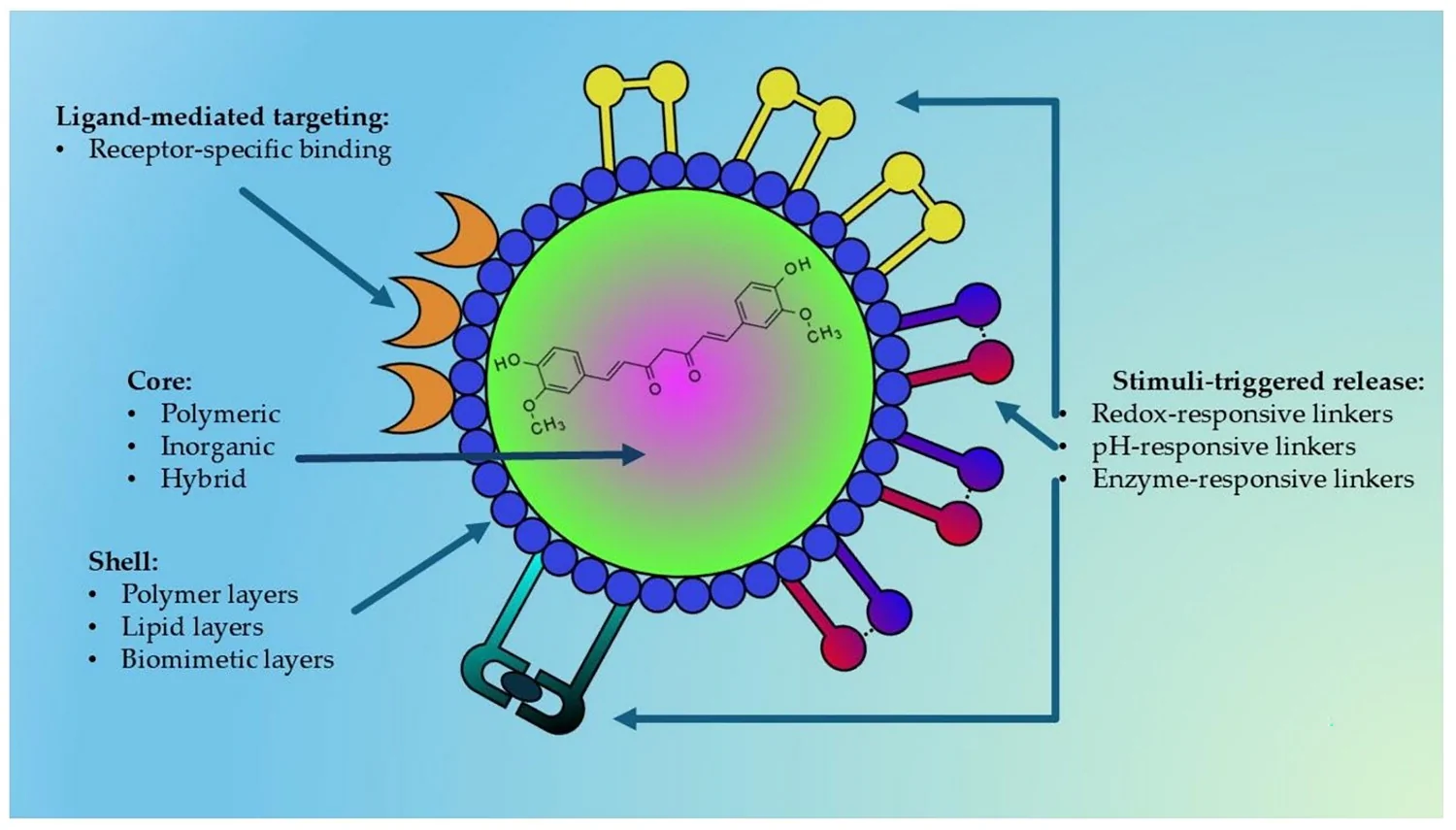
Diagnostic scheme of a NP that will deliver natural bioactive compounds (e.g. curcumin). It has an inner core which is polymeric, inorganic or hybrid and is chosen depending on the physicochemical properties of the compound or extract that is being encapsulated. Active targeting using surface ligands and controlled drug delivery based on changes in pH, intracellular GSH or enzymatic activity using stimulus-responsive linkers can be achieved. The pharmacokinetics and site-selective release of natural compounds can be regulated in fine. Reproduced from Ref. [43] under the terms and conditions of the Creative Commons Attribution (CC BY) licence (https://creativecommons.org/licenses/by/4.0/).

#### 2.3.1. Single ligand for targeting the same receptors on two different cells

Utilizing a single ligand that can both bypass physiological and pathological barriers and specifically recognize cancer cell membranes is an effective tumour-targeting strategy. This approach is especially advantageous for organs protected by barriers such as the blood-brain barrier (BBB) and testicular blood barrier, where drug delivery is challenging. The BBB’s resistance to therapeutic agents and xenobiotics is largely due to complex interactions involving supporting cells like astrocytes and microglia, as well as tight junctions between endothelial cells [49]. As such, several methods have been developed to facilitate the translocation from bloodstream to brain parenchyma through the BBB [50,51]. Brain capillary endothelial cells facilitate the entry of various molecules into the brain via molecular transport receptors such as low-density lipoprotein receptor-related protein, insulin receptor and TfR. Dual-targeting NPs exploit this by being functionalized with ligands specific to two distinct receptors on cancer cell surfaces. For instance, NPs designed to target both the FAR and TfR have been developed for several malignancies. This dual-targeting approach enhances cellular uptake and improves therapeutic efficacy by enabling more precise drug delivery compared to single-target systems [51].

#### 2.3.2. Two different receptors on different cells each targeting a different dissimilar receptor

Effectively crossing multiple physiological and pathological barriers to reach tumour sites often necessitates the use of NPs functionalized with multiple ligands. TME is characterized by a remodeled ECM, diverse proteins, glycosaminoglycans, fibroblasts and immune cells that collectively promote cancer invasion and progression while shielding the tumour from immune surveillance, particularly within the brain. The dense ECM presents a significant barrier, impeding the penetration of targeted nanostructures into deeper tumour regions [52]. Designing a ligand-targeted DDS capable of effectively binding to and penetrating ECM could substantially enhance glioma therapy by improving the retention of therapeutics within TME. The ECM poses a significant barrier, often limiting the infiltration and efficacy of therapeutic agents in the TME [53].

### 2.4. Advantages over single-target approaches in gynaecological cancers

Despite advancements in surgery and chemotherapy, ovarian cancer continues to have poor survival rates, underscoring the urgent need for novel therapies. Immunotherapy has emerged as a promising approach by targeting the TME and enhancing anti-tumour immune responses. Clinical strategies under investigation include immune checkpoint inhibitors such as PD-1/PD-L1 antagonists (pembrolizumab, nivolumab, avelumab), vaccines and adoptive T cell therapies. These therapies aim to reactivate exhausted T cells and promote durable immune-mediated tumour control. Early-phase clinical trials in heavily pretreated ovarian cancer patients have demonstrated encouraging response rates (around 10–17%) and manageable safety profiles, although challenges like tumour heterogeneity, immune evasion and adverse events persist. Ongoing research continues to optimize these combinations to improve outcomes for ovarian cancer patients, making immunotherapy a highly promising frontier in gynaecologic oncology [54–56].

Dual targeting enables simultaneous inhibition of multiple pathways or antigens involved in tumour progression, enhancing therapeutic efficacy in the heterogeneous tumours commonly seen in gynaecological cancers. For example, bispecific antibodies bind two distinct tumour-associated antigens, improving treatment specificity by directing immune cells more precisely to cancer cells. Since cancer cells often develop resistance to single-target therapies, dual-targeting strategies—such as bispecific T-cell engagers that activate T-cells against two targets—can reduce resistance risk. This approach, successful in other cancers, is under investigation for gynaecologic malignancies and holds potential for improved outcomes in patients refractory to single-agent treatments [57]. Dual targeting can enhance selectivity for malignant cells over healthy cells. By focusing on two specific antigens that are uniquely expressed or overexpressed on cancer cells, these strategies can minimize off-target effects and reduce damage to normal tissues (Figure 3). This increased selectivity is crucial in gynaecological cancers, where preserving healthy tissue is essential for maintaining quality of life during treatment [58].

**Figure 3.**
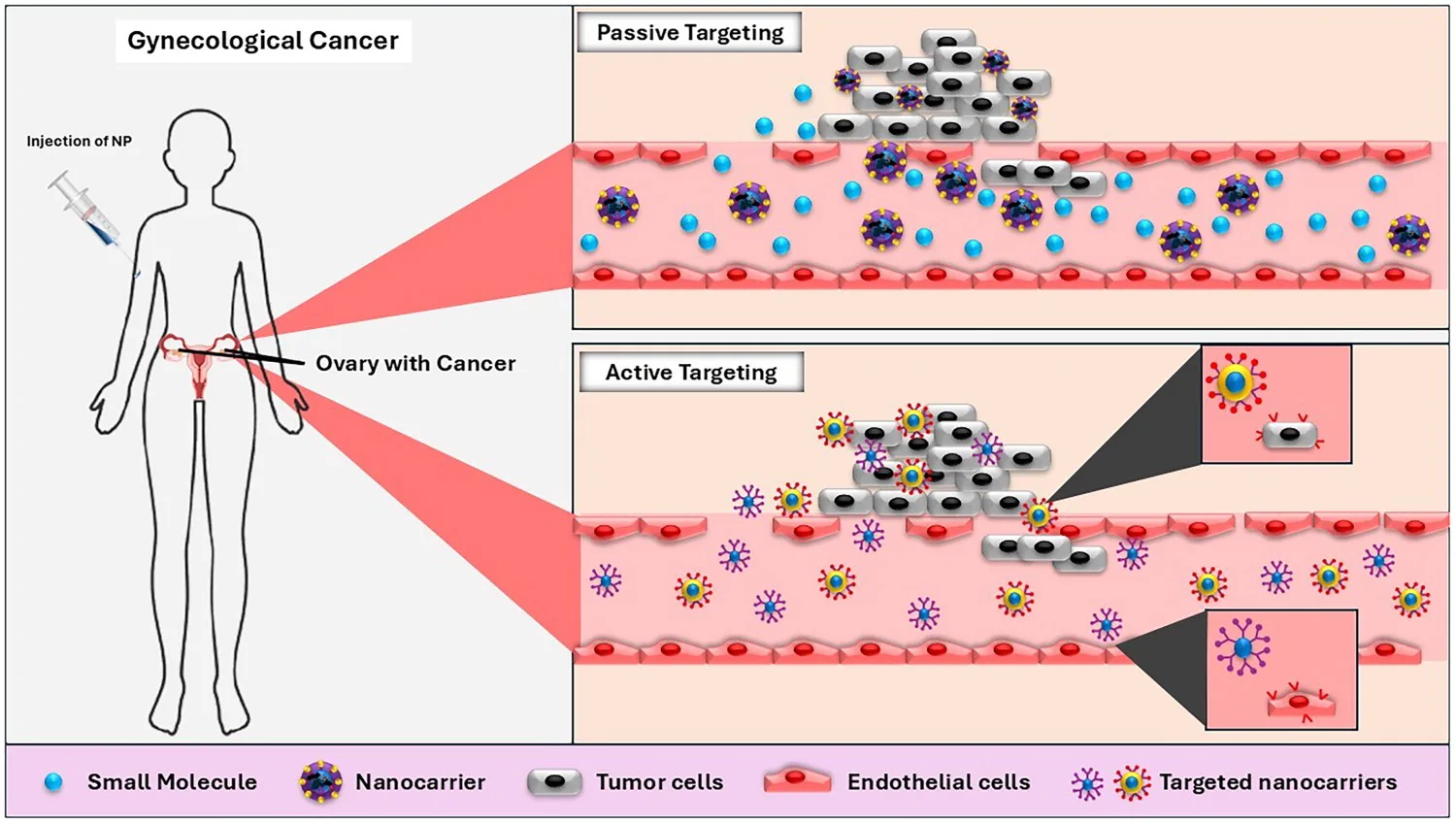
Targeting strategies in nanomedicine for gynaecological cancer treatment are illustrated. Passive targeting (upper panel) shows NPs being accumulated in tumour tissue by the EPR effect, but without specific targeting. In the lower panel, Active Targeting is displayed whereby functionalized nanocarriers are transported to tumour cells through ligands that bind directly to overexpressed tumour cell receptors for improved targeted delivery. The drugs are small molecules, whereas nanocarriers are used for targeted drug delivery. The authors themselves draw the figure.

## 3. Mechanisms of dual-targeted nanotechnology in gynaecological cancers

### 3.1. Mechanisms of tumour targeting

Active targeting nanomedicines exploit receptor-mediated endocytosis by binding to overexpressed receptors on tumour cells. However, single-ligand nanoparticulate systems face limitations due to TME complexity and tumour heterogeneity. Recently, dual-ligand nanomedicines have advanced tumour-targeted delivery by providing enhanced functional versatility, which improves the overall efficacy of these delivery systems [59]. Dual targeting is an emerging strategy that involves designing delivery systems functionalized with two distinct ligands, each targeting different receptors either on the same cell type or on different cell types. This approach aims to enhance the specificity of drug delivery to tumour cells by simultaneously engaging multiple cellular targets. This approach now explicitly includes function-driven therapeutic modalities such as targeted delivery combined with photothermal therapy, magnetic hyperthermia and theranostic imaging, enhancing precision beyond simple biomarker binding [60]. Recent advances demonstrate how dual-targeted systems can integrate emerging therapeutic modalities, such as photothermal therapy and magnetic hyperthermia, within theranostic frameworks. For example, magnetic NPs guided by external fields combined with photothermal conversion elements have shown enhanced tumour ablation and imaging capability, representing a dual-modal therapeutic paradigm that fuses diagnostic imaging with effective hyperthermic anti-tumour treatment. Dual-targeting nanomedicines combining magnetic guidance and photothermal effects have demonstrated synergistic multimodal therapy, where polydopamine-based NPs incorporate magnetic iron oxide cores for magnetic targeting along with innate photothermal properties to generate thermal ablation under near-infrared irradiation, resulting in stronger tumour inhibition than single-modal systems. For example, polydopamine-coated NPs (PDA-DEM-Fe_3_O_4_@M) showing enhanced tumour accumulation and synergistic therapeutic effects combining photothermal therapy and chemotherapy [48]. Additionally, hybrid HA/HSA nanocarriers loaded with DOX and ^131I enable dual-modal MRI/SPECT imaging while delivering chemotherapy, allowing real-time monitoring of drug distribution and therapeutic action simultaneously [61]. Theranostic nanocarriers have been developed that merge MRI with stimulus-triggered therapy. For instance, intelligent dual-responsive nanostructures encapsulating DOX and responsive metal oxide layers enable MRI-guided chemo-photothermal therapy and chemodynamic therapy, facilitating image-guided targeted release and synergistic cancer ablation *in vivo* [62]. Dual-targeted radiosensitizer agents have been investigated using AuNCs functionalized with FA and trastuzumab (Herceptin^®^) [63]. Additionally, trastuzumab was used as a chemotherapeutic agent in a variety of other cancer treatments, and as a bioligand to increase the internalization of NPs into HER2 positive breast cancer cells. This dual functionalization is aimed at increasing the delivery efficiency of therapeutic agents, especially to tumour cells [64,65].

A dual-targeted NPs (SSBPEI-DOX@siRNAs/iRGD-PEG-HA) was developed and targeted to deliver siRNA cocktails and DOX specifically to ovarian CSCs. Drug release in these NPs employs glutathione (GSH) responsive mechanisms, and they are functionalized with ligands that target neuropilin-1 and CD44, allowing them to deliver therapeutics directly to CSCs that are typically resistant to conventional treatments [13]. Researchers developed FA modified zeolitic imidazolate frameworks, FA-ZIF-90, for the delivery of cisplatin with an aim to overcome drug resistance in ovarian cancer cells, as illustrated in Figure 4. Enhanced targeting occurs with the FA moiety that binds specifically with FARs that are overexpressed on the surface of tumour cells, leading to the selective accumulation of the drug within the mitochondria of the tumour cells enhancing the therapeutic efficacy [44].

**Figure 4.**
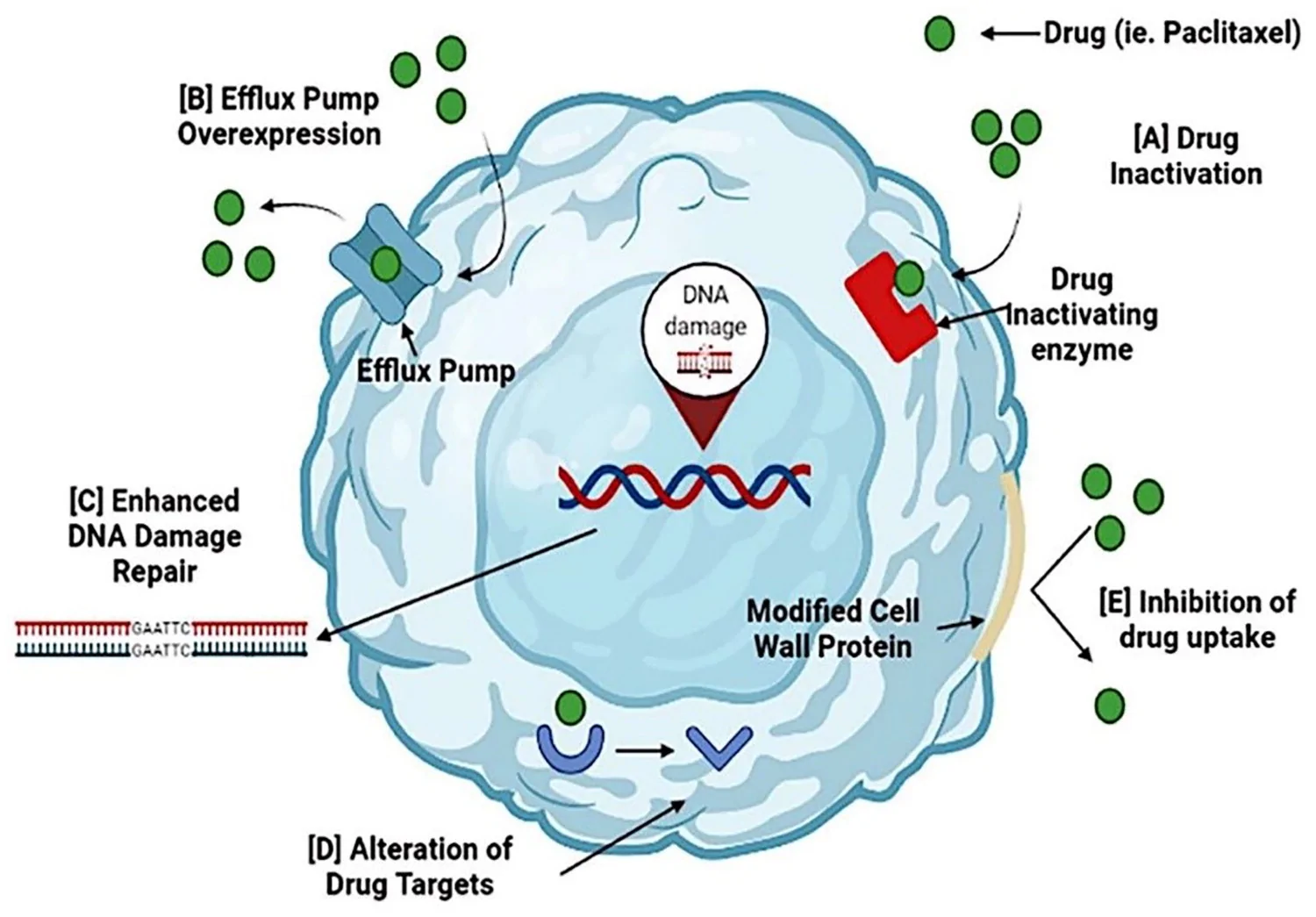
Drug resistance mechanisms in ovarian cancer. When there is chemotherapy, cancer cells can become resistant to the drug administered to them. There are several ways for resistance to chemotherapy. (Activation or activation of a drug) takes place, where the enzyme created code for resistance genes (MDR) that chemically modifies the drug. (A) The level of a drug in a cell is decreased by efflux pump overexpression; (B) efflux pump overexpression inhibits accumulation of a chemotherapeutic drug. DNA repair mechanisms, nucleotide excision repair and HR trigger (C) enhanced DNA damage repair. They reverse drug damage and increase drug resistance. (D) Chemotherapeutic drugs have specific targets so that changes in drug targets can alter the drug and make it ineffective. (Modifications in the cell wall proteins inhibit drug uptake into the cell) (E) due to inhibition of uptake of drug into cell. Cancer cells can make themselves sensitive to those drugs through the targeting of these resistance mechanisms. Reproduced from Ref. [66] under the terms and conditions of the Creative Commons Attribution (CC BY) licence (https://creativecommons.org/licenses/by/4.0/).

### 3.2. Synergistic effects of dual targeting

A study developed dual-ligand NPs for delivering siRNA targeting P21-activated kinase 1 (PAK1) in hepatocellular carcinoma. The NPs were functionalized with galactose and glycyrrhetinic acid to enhance targeting. This dual-targeting strategy significantly increased cellular uptake of siPAK1, resulting in reduced cell proliferation, invasion and migration, alongside increased apoptosis. *In vivo*, this approach demonstrated superior anti-tumour efficacy in tumour-bearing mice, highlighting the therapeutic potential of dual-targeted nanocarriers [67]. Dual-targeted hybrid NPs conjugated with the ArgGlyAsp (RGD) peptide, co-loaded with DOX and mitomycin C, were developed towards treating lung metastases of triple negative breast cancer. *In vitro*, these NPs effectively targeted both tumour vasculature and cancer cells, resulting in increased cytotoxicity. In a murine model, the dual-targeting approach significantly reduced lung metastasis burden and extended median survival compared to free drugs or non-targeted formulations [68]. Another study highlighted the benefits of NP-mediated co-delivery of multiple therapeutic agents to overcome the limitations of single-drug treatments. Combining cytotoxic drugs with nucleic acids in NPs enabled simultaneous targeting of multiple pathways, significantly enhancing tumour inhibition. This strategy optimized drug efficacy while minimizing systemic side effects, offering both synergistic and drug-specific therapeutic effects [69].

Recently, a dual-targeting nanomedicine demonstrated synergistic multimodal therapy by combining photothermal therapy with chemotherapy. The integration of photothermal and chemotherapeutic agents within a targeted delivery system produced a potent inhibitory effect on tumour growth, underscoring the effectiveness of combining diverse therapeutic modalities [70]. Recent studies have demonstrated the synergistic potential of combining the poly (ADP-ribose) polymerase (PARP) inhibitor niraparib with DOX for ovarian cancer treatment. Liposomal formulations encapsulating both agents and functionalized with folate enable selective targeting of the folate receptor alpha, which is overexpressed on ovarian cancer cells. Targeted delivery of DOX is enhanced through receptor-mediated endocytosis. The observed synergistic interaction between niraparib and DOX is dependent on the molar ratio of the two drugs and varies with the ovarian cancer cell line. Notably, a specific 1:1 molar ratio consistently produced synergistic or additive effects across homologous recombination (HR)-deficient cell lines, whereas in the HR-proficient PEO4 cell line, the same ratio exhibited antagonistic effects. These findings suggest that HR-proficient tumours may derive limited benefit from the DOX and PARP inhibitor combination [71].

## 4. Clinical applications of dual-targeted nanotechnology in gynaecological cancers

Dual-targeted nanotechnology has shown recent promise in taking gynaecological cancer treatment to the next level with a high degree of therapeutic precision and low risk of systemic toxicity. Recent studies show dual-targeted NPs have been successfully used for targeted drug delivery, gene therapy and immunotherapy of ovarian, cervical and endometrial cancers, as illustrated in Table 1.

**Table 1.** Summary of clinical and preclinical dual-targeted nanotechnology systems in gynaecological cancers.

Type of cancer	Nanotechnology system	Stage	Key findings	Ref.
Ovarian	PLD (Doxil/Caelyx)	Approved	FDA/EMA approved, improved tolerance vs monotherapies, combination with carboplatin improves PFS, reduced neuropathy and hair loss	[72]
Ovarian	OLICARB (PEGylated liposomes co-encapsulating olaparib + carboplatin)	Preclinical	Enhanced cytotoxicity (3–13×), synergistic effect, selective tumour targeting, reverses MDR	[73]
Ovarian	FA-targeted Fe_3_O_4_ NPs	Preclinical	T2 MRI contrast, low toxicity, tumour-specific imaging and potential therapy	[74]
Cervical	TAT-PTX/TOS-CDDP SLNs	Preclinical	Co-delivery of paclitaxel + cisplatin, synergistic tumour inhibition, targeted accumulation, reduced systemic toxicity	[75]
Cervical	5-FU loaded SLNs in TNOs	Preclinical	Stimuli-responsive, precise cervical tissue release, enhanced therapeutic potential	[76]
Cervical	Curcumin-modified AuNPs for IL-12 delivery	Preclinical	Stable delivery, good cytocompatibility, high IL-12 expression, cytokine-based immunotherapy	[77]
Endometrial	FA + HA dual-targeted NPs delivering paclitaxel	Preclinical	Enhanced tumour accumulation, improved specificity, reduced systemic toxicity	[78]
Vulvar	Dual-targeted CRISPR/Cas9 NPs	Preclinical	Tumour-specific gene editing, reduced off-target effects, smaller tumour size, higher apoptosis	[79]
Peritoneal carcinomatosis	Targeted micelles co-delivering paclitaxel + immune checkpoint inhibitor	Preclinical	Localized drug accumulation, synergistic anti-tumour effect	[80]
Uterine sarcoma	Liposomes delivering DOX + siRNA	Preclinical	Tumour-specific uptake, reduced tumour growth, improved survival in animal models	[81]
Endometrial	CNSI contrast agent for SLNB	Clinical	Higher detection rate and accuracy with cervical injection, safe and effective	[82]

### 4.1. Ovarian cancer

In ovarian cancer, first-line therapy commonly involves the use of combination anticancer drugs and small-molecule therapeutic agents with different mechanisms of action, often delivered via nanoparticulate systems. A major challenge limiting the efficacy of treatment is chemotherapy resistance, which is frequently associated with aberrant gene expression. For instance, reduced sensitivity of cancer cells to anticancer drugs has been linked to the upregulation of genes such as MDR1 and Bcl-2 [83]. Thus, the combination of chemotherapy with siRNA-based gene therapy has been shown to effectively reverse MDR in ovarian cancer [84,85]. The high efficacy of the PARP inhibitor olaparib combined with the platinum-based anticancer drug carboplatin was demonstrated in a formulation termed OLICARB, where both drugs were co-encapsulated in PEGylated liposomes. This dual-DDS improved therapeutic outcomes in ovarian cancer treatment. Experiments were conducted on two ovarian cancer cell lines: A2780 (cisplatin-sensitive) and A2780cis (cisplatin-resistant), using varying molar ratios of olaparib to carboplatin (OLICARB 1:1 and 2:1). Results showed that encapsulating olaparib and carboplatin within PEGylated liposomes significantly enhanced the cytotoxicity of the drug combinations compared to free drugs, with fold increases in toxicity ranging from 3 to 13 depending on the molar ratio. The cisplatin-resistant A2780cis cells exhibited the most pronounced response. Combination index analysis further confirmed that OLICARB exhibited a stronger synergistic effect than free drugs or individual liposomal drug formulations. Additionally, OLICARB NPs demonstrated marked selectivity for tumour cells over normal cells, single-arm preclinical studies, not yet tested in randomized clinical trials. The anti-tumour efficacy was correlated with increased DNA damage, as olaparib potentiated carboplatin toxicity by inhibiting DNA repair mechanisms responsible for repairing carboplatin-induced cytotoxic DNA lesions [86].

PEGylated liposomal doxorubicin (PLD), marketed as Doxil (US) and Caelyx (Europe/Canada), was the first FDA-approved nanodrug for ovarian cancer and multiple myeloma. It gained FDA approval in 1999 for advanced ovarian cancer patients resistant to platinum-based therapy, with EMA approval following in 2000 [87,88]. A meta-analysis of randomized trials showed PLD has similar efficacy but better tolerance compared to other monotherapies in ovarian cancer. Combining carboplatin with PLD improved progression-free survival (PFS) but not overall survival versus carboplatin-paclitaxel. This combo caused less neuropathy, hair loss and neutropenia but more gastrointestinal toxicity, anaemia and skin reactions [89].

Targeted therapies using antibodies against HER2 have shown limited responses in clinical trials with patients, because the overall expression of HER2 in ovarian cancer is lower compared to HER2 positive breast cancer. Average HER2 positive tumour cell content in human ovarian cancer tissues is 10–20%. Thus, current research efforts are aimed at the development of optimal strategies utilizing NPs delivery systems [90,91]. Most of these approaches are still in preclinical or early-phase clinical studies, and randomized trial data are not yet available. FA-targeted iron oxide (Fe_3_O_4_) NPs have been developed as T2 negative MRI contrast agents to accurately detect ovarian tumours in xenograft models. Their low toxicity and biodegradability make them promising for early diagnosis and treatment of ovarian cancer [74].

### 4.2. CC

NP-based techniques offer greater selectivity and sensitivity than traditional diagnostics. Using nanomaterials like gold NPs, their unique optical and electrical properties enable advanced sensors such as electrochemical and DNA biosensors to detect biomarkers qualitatively and quantitatively [92]. The key advantage of this strategy is its ability to improve low conductivity, low sensitivity and high costs associated with conventional sensors, effectively overcoming their limitations [93,94]. Liu and colleagues explored the co-delivery of paclitaxel and TOS-cisplatin via trans-activating transcriptional activator (TAT)-modified SLNs, showing synergistic anti-tumour effects against CC. The TAT-PTX/TOS-CDDP SLNs were efficiently internalized by HeLa cells preclinical trials, significantly inhibiting tumour cell proliferation. *In vivo* studies demonstrated targeted tumour accumulation, strong anti-tumour efficacy and reduced systemic toxicity. These findings highlight the potential of dual-drug SLN delivery as an effective therapeutic strategy for CC and possibly other malignancies [75].

SLNs, known for their high bioavailability and low toxicity, offer a promising strategy for targeted drug delivery. A novel approach using SLNs encapsulating 5-fluorouracil (5-FU), integrated into various thermo-sonic nano-organogel (TNO) systems, was developed for stimuli-responsive therapy of CC. By incorporating PLA, palmitic acid and polyvinyl alcohol into nanospherical SLNs within TNOs and activating them with thermal and ultrasound triggers, targeted release of 5-FU was successfully achieved exclusively in cervical tissue, demonstrating high precision and therapeutic potential [76]. Curcumin-modified gold nanoparticles (AuNPs) have been shown to significantly enhance the delivery of interleukin-12 (IL-12) in an *in vitro* model of CC. These stable, spherical AuNPs effectively condensed and protected IL-12 DNA while maintaining good cytocompatibility. The system achieved substantial IL-12 expression, highlighting its potential as a promising platform for cytokine-based immunotherapy in CC treatment [77]. Nanotechnology provides highly potential in treating CC, and it also provides strong potential of interdisciplinary collaboration, integrating insights from different areas to enhance developments and creations [95].

### 4.3. Other gynaecological cancers

Dual-targeted NPs utilizing FA and hyaluronic acid (HA) have been developed for the selective delivery of paclitaxel to endometrial cancer cells. FA targets cancer cells overexpressing FARs, while HA binds to CD44 receptors, both of which are commonly upregulated in endometrial cancer. This dual-ligand approach enhances cellular uptake and therapeutic efficacy by improving specificity and reducing off-target toxicity [78]. This dual-targeting strategy demonstrated enhanced drug accumulation within tumour tissues and reduced systemic toxicity, resulting in improved therapeutic outcomes compared to conventional delivery methods. Additionally, a novel approach has been developed employing dual-targeted NPs to deliver CRISPR/Cas9 systems targeting oncogenes specific to vulvar tumours, offering a promising gene therapy platform for the treatment of vulvar cancer [79]. Specifically, these NPs are designed to bind to cancer cell receptors to entice the gene editing, minimizing off-target effects. Initial studies demonstrate reduced tumour size along with higher apoptosis rates of cancer cells [13].

For the treatment of peritoneal carcinomatosis arising from gynaecological cancers, targeted micelles were utilized for the co-delivery of paclitaxel and an immune checkpoint inhibitor. These micelles were functionalized with ligands that specifically bind to peritoneal tumour cells, facilitating localized drug accumulation at the tumour site while minimizing systemic exposure. Preclinical studies demonstrated that this combinatorial approach produced synergistic anti-tumour effects, highlighting its potential as a promising therapeutic strategy [80]. In uterine sarcoma, liposomal systems have been designed to co-deliver DOX and siRNA targeting anti-apoptotic genes. These liposomes are functionalized with tumour-specific peptides that bind selectively to tumour-associated antigens, promoting preferential uptake by cancer cells while minimizing effects on healthy tissues. Preclinical studies in animal models have shown a significant reduction in tumour growth and improved survival outcomes, underscoring the potential of this targeted therapeutic approach [81]. In clinical context, Zuo et al. conducted a prospective consecutive study to evaluate the effectiveness of a cervical and fundal injection of the contrast agent CNSI for facilitating sentinel lymph node biopsy (SLNB) mapping during laparoscopic surgery in patients with endometrial carcinoma. Fifty patients were treated with fundal sub-serosal injections at four sites (fundal group) and 65 patients were treated with cervical sub-mucosal injections at two sites (cervical group). SLNB mapping using CNSI in laparoscopic procedures for endometrial cancer was found to be both safe and effective, with cervical injection resulting in higher detection rate and greater accuracy than fundal injection [96,97].

## 5. Challenges and limitations in dual-targeted nanotechnology for gynaecological cancers

### 5.1. Biocompatibility and toxicity (material and biological challenges)

Nanocarriers, including liposomes, polymeric micelles and dendrimers, have shown promise in the treatment of gynaecologic cancers such as ovarian, cervical and endometrial cancers. Liposomes, for instance, can encapsulate both hydrophilic and hydrophobic drugs, enhancing drug solubility and stability. However, their clinical application is limited due to challenges in drug-loading capacity, rapid clearance by the MPS and complex multi-ligand formulations that hinder scalability, leading to reduced therapeutic efficacy. Polymeric micelles offer improved stability and controlled drug release, but their clinical translation is hindered by issues related to scalability, potential toxicity and complex synthesis protocols. Dendrimers, with their well-defined structure, allow for precise drug loading and surface modification, yet concerns regarding biocompatibility, immunogenicity and reproducibility remain critical. Moreover, the heterogeneity of gynaecologic tumours, including variations in vascular permeability and TME, poses additional challenges in achieving effective drug delivery. Therefore, while nanocarriers hold significant potential, their clinical success in gynaecologic oncology requires addressing these multifaceted material- and biology-related challenges through continued research and development [80,98–100].

The materials used to fabricate NPs can significantly influence their biocompatibility. For instance, gold NPs are noted for excellent biocompatibility but may have low drug loading and limited tumour penetration, whereas carbon nanotubes, while offering high drug loading, are associated with high toxicity and limited biocompatibility [101,102]. Surface chemistry and ligand functionalization can enhance targeting but may also induce immunogenicity or toxicity if not optimized. While targeted therapies aim to minimize collateral damage to healthy cells, systemic NP administration can lead to unintended interactions with non-target tissues, particularly relevant in gynaecologic cancers [103]. Cancer cells often develop resistance even to NP-based therapies, complicating treatment and potentially requiring higher drug doses, thereby elevating systemic toxicity. Precise spatiotemporal control over drug release from NPs is critical, yet many current formulations lack robust *in vivo* control, limiting clinical effectiveness [13]. Regulatory guidelines mandate comprehensive nanotoxicological evaluation of nanomedicine formulations at early stages of clinical development. This involves the use of validated and sensitive assay protocols across *in vitro*, *ex vivo* and *in vivo* models. Such assessments are essential to ensure the safety, efficacy and biocompatibility of nanomedicines before advancing to later-phase clinical trials, ultimately supporting informed risk–benefit decisions [104,105]. Because nanoencapsulation often prolongs circulation half-life and drug retention, specialized toxicology studies in animal models are essential to evaluate both short- and long-term toxic effects. Accurately predicting *in vivo* toxicological responses requires a thorough understanding of how nanomaterials are absorbed, distributed, metabolized and excreted (ADME) within the body [104,106].

### 5.2. Clinical translation (biological and regulatory challenges)

The conventional paradigm of nanomedicine development has been formulation-based where challenges in clinical translations turn out to be evident during the late stages of development. Biological factors, disease pathophysiology and patient heterogeneity should be learned to enhance success and NP accumulation, distribution, retention and efficacy. It is necessary to define biopharmaceutical correlations between the properties of delivery system and *in vivo* behaviour. The clinical translation of nanomedicines is restricted even though a substantial research has been carried out in the field. A large part of cancer-targeting nanomedicine is based on the EPR effect, which is only seen in a few types of tumour and lacks consideration of heterogeneity of tumours in patients, which is a limitation to success [107–110]. Optimal targeting ligand density is underexplored, with intermediate density suggested to improve targeting; detailed studies linking ligand density to target expression and internalization are needed [111].

### 5.3. Manufacturing and scale-up (material and regulatory challenges)

A major obstacle to clinical translatability of nanomedicine formulations is their structural and physicochemical complexity. While industrial-scale production methods exist for basic nanomedicine platforms like liposomes, adding complexity such as surface coatings, multiple ligands or co-encapsulation of drugs requires multi-step manufacturing, raising costs and complicating quality assurance and regulatory compliance, posing barriers to clinical translation [112]. Regulatory guidelines mandate comprehensive nanotoxicological evaluation at early stages of development, including ADME, short- and long-term toxicity studies, to ensure safety, efficacy and biocompatibility before advancing to clinical trials [106,113].

## 6. Future directions and emerging trends

Current cancer treatments often suffer from limited effectiveness and significant side effects, making sustained remission challenging. Looking ahead, nanotechnology offers promising opportunities to transform cancer diagnosis and therapy [114]. Advances in nanomaterials are enhancing DDSs by developing novel nanocarriers such as lipid-based, polymeric and inorganic NPs. These materials aim to improve drug solubility, bioavailability and targeted delivery to tumours while minimizing side effects of traditional therapies. Emerging strategies like hierarchical targeting—simultaneously directing therapeutics to tissue, cells and organelles—enable more precise treatment with fewer off-target effects. Functionalizing nanocarriers with ligands that bind tumour-specific antigens or receptors further boosts cellular uptake and therapeutic efficacy [115].

Clinically, dual-targeted nanotechnology is being considered as an enabling technology that can be incorporated with the current standards of care and not as a replacement of it. DTN systems have the potential to maximize neoadjuvant therapy of ovarian cancer by enhancing intratumoural drug delivery and combating chemoresistance before cytoreductive surgery and by helping with maintenance treatment due to sustained and local drug delivery with less systemic toxicity. In CC, DTN systems have the potential to be used in combination with chemoradiotherapy as an opportunity to enhance radiosensitization and spatial drug localization, which is likely to better manage the tumour and spare the adjacent healthy tissues. DTN can be optimally placed in the adjuvant context of endometrial cancer where surgery is the primary modality of treatment to either suppress recurring disease or provide therapy with enhanced specificity. These disease-specific integration approaches underscore the future opportunities of customizing DTN to specific clinical processes in gynaecologic malignancies such that it can be integrated into multimodal cancer therapies in the future [116].

Incorporation of artificial intelligence (AI) into nanocarrier development has emerged as a powerful approach to optimize NP design, predict biological interactions and improve therapeutic efficacy. AI tools such as machine learning and deep learning can analyse complex datasets to guide rational design of nanocarrier properties (size, surface charge, ligand density) and predict drug delivery performance, thereby accelerating development and reducing reliance on trial-and-error methods. AI-aided nanocarrier design also plays an important role in theranostics by predicting optimal integration of imaging agents within delivery systems and modelling drug release behaviours under physiological conditions [117]. Although most work to date is in general oncology models, these computational approaches set the foundation for future AI-enhanced nanocarrier design in gynaecological cancers as data and clinical evidence continue to grow.

## 7. Conclusion

Substantial advancements have been made in the field of gynaecologic oncology through the exploration of nanotechnology-based DDSs and innovative targeting strategies, which have demonstrated significant potential to improve therapeutic efficacy and reduce off-target effects. However, challenges remain in translating these promising preclinical results into consistent clinical success due to complex TMEs and biological barriers. Current evidence suggests that dual-targeted systems based on clinically established nanoplatforms using ligands targeting FARs, CD44 or tumour vasculature promising directions for near-term clinical translation. Combination strategies integrating chemotherapy with gene therapy or immunomodulation within these platforms appear especially relevant for addressing chemoresistance in ovarian cancer and CC. AI offers complementary opportunities to enhance diagnostic accuracy, optimize treatment planning and monitor patient responses, although its clinical implementation requires validation, data privacy safeguards and integration into existing healthcare frameworks. Multidisciplinary approach combining nanotechnology, AI and rigorous clinical research is essential to advance personalized medicine in gynaecologic cancers and improve patient outcomes.

## Data Availability

Not applicable. This is a review article, and all relevant information is provided in the article.
